# An Automatic Multi-Target Independent Analysis Framework for Non-Planar Infrared-Visible Registration

**DOI:** 10.3390/s17081696

**Published:** 2017-07-26

**Authors:** Xinglong Sun, Tingfa Xu, Jizhou Zhang, Zishu Zhao, Yuankun Li

**Affiliations:** 1 Image Engineering & Video Technology Lab, School of Optoelectronics, Beijing Institute of Technology, Beijing 100081, China; 2120545@bit.edu.cn (X.S.); xiaomianzhou@126.com (J.Z.); nicholasldm@126.com (Z.Z.); liyuankunbixian@gmail.com (Y.L.); 2 Key Laboratory of Photoelectronic Imaging Technology and Systems, Ministry of Education of China, Beijing 100081, China

**Keywords:** multi-target registration, infrared-visible videos, non-planar, feature matching, Gaussian criterion, multi-target tracking

## Abstract

In this paper, we propose a novel automatic multi-target registration framework for non-planar infrared-visible videos. Previous approaches usually analyzed multiple targets together and then estimated a global homography for the whole scene, however, these cannot achieve precise multi-target registration when the scenes are non-planar. Our framework is devoted to solving the problem using feature matching and multi-target tracking. The key idea is to analyze and register each target independently. We present a fast and robust feature matching strategy, where only the features on the corresponding foreground pairs are matched. Besides, new reservoirs based on the Gaussian criterion are created for all targets, and a multi-target tracking method is adopted to determine the relationships between the reservoirs and foreground blobs. With the matches in the corresponding reservoir, the homography of each target is computed according to its moving state. We tested our framework on both public near-planar and non-planar datasets. The results demonstrate that the proposed framework outperforms the state-of-the-art global registration method and the manual global registration matrix in all tested datasets.

## 1. Introduction

Nowadays there is considerable interest in multi-sensor fusion, particularly infrared-visible sensor fusion [1,2,3]. This is because these sensors can provide complementary information for scenario analysis. Many applications, ranging from human detection [4], visual surveillance, and target tracking to medical imaging [5] can benefit from this fusion. At this time, registration is required to align the images (or videos) captured by different sensors, which is a very important step to achieve image fusion. Therefore, this paper focuses on studying infrared-visible video registration with multiple targets on non-planar scenes (i.e., scenes in which these targets lie on different depth planes).

In previous works, various approaches have been introduced to solve the infrared-visible image registration problem, such as area-based methods [6,7,8] and feature-based methods [9,10,11,12]. These works led to some progress in improving registration quality or reducing computational time, but there are still some difficulties that need to be overcome. Area-based methods adopt area information to find a transformation, but they are not well suited for infrared-visible registration, since these two kinds of images will manifest different information of a scene [7]. Furthermore, if the images are not rectified [8], it will be difficult to precisely align all targets on non-planar scenes in these methods. In consideration of these facts, we choose featured-based methods for accurate registration.

Feature-based methods extract a variety of features for image registration. Though the texture information between infrared-visible images is inconsistent, some methods [10,11,12] can still find reliable features. These methods not only can process the planar scene, but also can find a frame-wide homography for the near-planar scene (in which targets almost lie in the same depth plane). However, they also have some drawbacks. First, they deal with all targets at a single frame together to find matches [10,12]. Therefore, they usually have a quite high computational complexity. Besides, the global matching strategy may introduce more outliers and reduce the quality of matches. Second, the depth differences between targets are very obvious in many observed scenes (non-planar scenes). These existing methods [10,11,12] cannot align the scenes, since one global homography is not enough to register the targets on different depth planes, even if it is a frame-wide homography.

To address the above problems, we propose an accurate registration framework. For the targets on non-planar scenes, more than one matrix is required to register them. Taking into account this fact, our framework achieves non-planar multi-target registration by estimating a homography for each target. During this process, we don't need to consider the depth values of the targets. More precisely, we first present a novel feature matching strategy. In the strategy, the foregrounds are matched. The results are utilized to constrain the feature matching. Then, a reservoir is created for each target to save the corresponding matches, and a multi-target tracking method [13] is adopted to distinguish different reservoirs. The transformation of each target is computed with all matches in its reservoir. The proposed method is very appropriate for visual surveillance, especially when the distance of monitoring is frequently varying. In practice, target tracking [14] has been applied in the registration domain. They are typically used to extract trajectories for registration. However, the purpose of performing tracking in our framework is different from previous works. As in some registration methods, we suppose that there are moving objects on the observed scenes, which are synchronized in the infrared-visible videos. The significant contributions presented in this paper may be summarized as follows:

First, we propose a novel automatic registration framework for infrared-visible videos, which registers every target. The proposed method can implement accurate multi-target registration on non-planar scenes.

Second, a new feature matching strategy is presented to find correspondences, which introduces a simple foreground matching algorithm to guide the feature matching. The strategy is faster and more robust than global matching strategies.

Third, we adopt a multi-target tracking method to distribute a reservoir for each target in the current frame. For targets with different motion states, their reservoirs are assigned using different methods. This way, sufficiently reliable feature pairs are provided to estimate a frame-wide homography for each target.

The rest of this paper is organized as follows: related work in the domain is explored in Section 2. In Section 3, we present our proposed method. In Section 4, we provide a description of our experiments and summarize the results, followed by our conclusions in Section 5.

## 2. Related Work

The key step of registration is to find correspondences between images (or videos). Most approaches have been developed to implement this step. These methods are generally studied for homologous sensor pairs, such as visible stereo or remote sensing pairs. It is not straightforward to apply them to infrared-visible pairs, since the pairs reflect different phenomena. Infrared sensors record infrared radiation emitted by objects, while visible sensors record light reflected from objects. Next, we briefly review two types of registration methods: area-based methods and feature-based methods.

### 2.1. Area-Based Methods

Some previous methods developed a few similarity functions to directly measure the relevance of the image region, such as region correlation [6] and mutual information [7,15,16]. For the methods using region correlation, they first compute the cross-correlation of each window pair in two images, and then view the pair with the maximum value as a correspondence. There are some problems for these methods, like being unreliable in textureless areas and time-consuming. The mutual information could evaluate the information dependence by calculating the entropy of each image [15]. In infrared-visible image registration, it was usually applied only on a selected part of images, such as the boundaries [16] and the foregrounds [7]. This is because there are obvious differences in intensity and texture between two sources.

Other area-based methods convert images into the frequency domain using Fourier transform and then calculate correlations [17]. Compared to region correlation methods, they are relatively faster and more robust to the noise. Actually, the above area-based methods could align planar scenes, but it is hard to realize precise non-planar scene registration using them. The method presented in [8] is able to align non-planar scenes using region information. However, it only can process the video pairs which have been rectified, which are not available.

### 2.2. Feature-Based Methods

As the simplest features, points have been universally applied in registration, and a variety of algorithms have studied how to match feature points [9,18]. However, extracting features directly from infrared-visible images is unreliable because of the texture difference. The boundaries are a popular solution since they are usually captured by both sensors. Therefore, most scholars adopt edges [19] or features on edges [20]. In their work, the edge information was aligned with different ways, such as Gaussian field criterion [21]. However, unprocessed edges are unreliable and easily influenced by noise.

In recent years, foreground detection has been adopted to improve the precision in feature extraction. Here, some methods adopt tracking to take advantage of detected foregrounds [22,23,24]. They extract the trajectories of all targets, and then use these trajectories to estimate a frame-wide homography for the whole scene. Other approaches directly find features on the contours of foregrounds [10,11,12]. A reservoir is then applied to save matches from different frames. The reservoir may be updated by different strategies, such as First in, First out (FIFO) [10] or voting based on a RANSAC algorithm [12]. These methods also find only one frame-wide homography. Actually, these kinds of methods are proposed to align planar or near-planar scenes. Nevertheless, a matrix is insufficient to align multiple targets on different depth planes (but it is enough for one target, even if the movement of the target is non-planar). As a result, they are not suitable for non-planar scene registration. The work in [11] present the idea of considering each target individually, but it does not introduce the approach of matching targets in a complex scene, and still computes only a homography for all targets.

The proposed method is related to the work of [10,11,12], but we make some important contributions to achieve non-planar registration in infrared-visible videos. In our work, we present a simple foreground matching algorithm to improve the accuracy of feature pairs. Then, we determine a frame-wide homography for every target based on a multi-target tracking method [13]. These contributions make our proposed framework more precise and have a more extensive range of applications.

## 3. Proposed Framework

**Figure 1:**
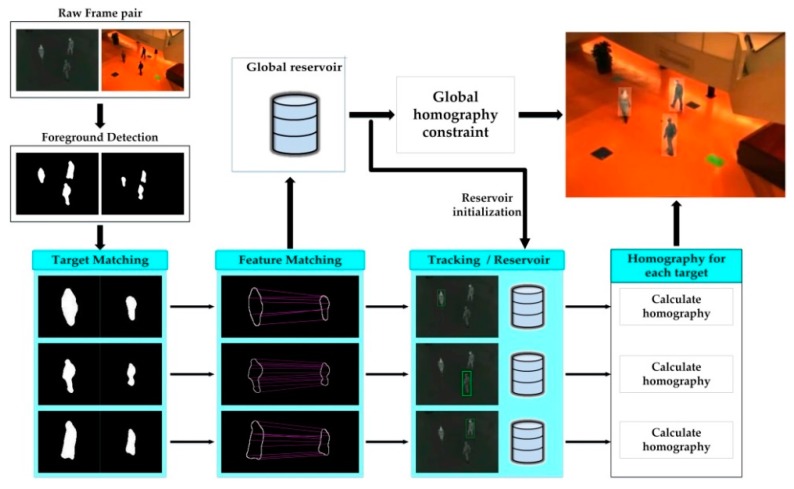
Overview of our proposed framework.

Figure 1 illustrates the processes of our method. First, a foreground detection algorithm [25] is applied to get foregrounds from raw image pairs. We extract feature points for registration from the contour of each foreground. Next, we match the infrared and the visible foregrounds based on two shape context descriptors [26]. Feature matching is then performed according to the foreground correspondences. For aligning non-planar scenes, we assign a reservoir for each target. Under the circumstances, multi-target tracking [13] is needed to distinguish different reservoirs. Following that, the homography of a target is estimated with all matches in its assigned reservoir. For ensuring the registration accuracy, we also create a global reservoir to save matches from all targets, and then compute a global frame-wide homography. They are used to initialize the newly created reservoir and constrain the registration of each target, respectively. In the subsequent subsections, we thoroughly introduce each step of the proposed framework.

### 3.1. Foreground and Feature Extraction

This work focuses on registering moving targets on static scenes, so the moving target detection is the initial step of our method. The PAWCS algorithm [25] is executed to subtract backgrounds, which builds a statistical background model using color and binary features, and applies a feedback scheme to identify foregrounds. At this time, morphological operations such as closing and hole filling are adopted to get improved candidate foreground blobs. The foreground blobs whose areas are relatively very small are abandoned. To solve the foreground fragmentation problem, when two blobs are very close (smaller than a fixed distance $D_{c}$), and the ratio of their areas (small/large) is smaller than a threshold $T_{a}$, they are merged together. After these, more reliable foregrounds can be obtained. Since some deviations may occur during foreground detection, we do not adopt all contour points of foregrounds for registration. The Curvature Scale Space (CSS) corner detection algorithm [20] is used to extract features from contours, which views the contour point with the curvature maximum as a feature. The algorithm can locate features accurately with a fast computational rate.

### 3.2. Feature Matching

For infrared-visible image registration, finding feature correspondences is an important and challenging step. Previous approaches [10,12] mostly adopt the global matching strategy, in which all features from multiple targets are matched simultaneously and globally. Indeed, the correspondences should be found only on the corresponding blobs. Therefore, this section presents a novel algorithm to process feature matching, which is based on the corresponding relations between foregrounds.

In intersecting fields, the shape information of targets and the spatial relationships between objects are mostly preserved. Based on this observation, we can match infrared and visible foregrounds by using two descriptors. For a foreground blob, its centroid is first computed. We build the first shape context descriptor with its own contour points using the method in [26], which expresses the disposition of contour points relative to the centroid using a uniform log-polar histogram. The descriptor utilizes the shape information. The second shape context descriptor is obtained using the contour points from other foregrounds, which displays the relative spatial distributions between targets.

After describing all foregrounds in infrared and visible images, the $\chi^{2}$ text statistic is used to measure the similarity between two descriptors, which is defined as:

(1) $$C\left(S_{i}\left(k\right),S_{v}\left(k\right)\right)=\frac{1}{2}\overset{K}{\underset{k=1}{\sum }}\frac{{\left(S_{i}\left(k\right)-S_{v}\left(k\right)\right)}^{2}}{S_{i}\left(k\right)+S_{v}\left(k\right)}$$

where $S_{i}\left(k\right)$ and $S_{v}\left(k\right)$ represent the K-bin normalized shape contexts of an infrared and a visible foregrounds, respectively. We accumulate the $\chi^{2}$ statistic values of two kinds of descriptors as the cost metric of foreground matching. For an infrared foreground, the visible foreground with minimal cost metric is deemed to be its candidate correspondence. The process is executed from visible to infrared foregrounds too, since foreground mismatch would seriously reduce the quality of feature correspondences. Only when a visible foreground is the candidate correspondence of an infrared foreground and vice versa, they would be regarded as a matched pair.

Like for the foreground, every feature point is described by a shape context using all contours in the image. Equation (1) is also used to measure the descriptor similarity. Then, we match the features from each foreground individually. For a foreground in the first image modality, we first consider if its corresponding blob existed in the second modality. If it existed, feature correspondences are found only on the corresponding blob pair; if not, we find matching points on all foregrounds in the second modality. The correspondence problem is solved using the Hungarian algorithm [27]. For the matches from various targets, they would be saved in different reservoirs, which is detailed in Section 3.3.

### 3.3. Reservoir Creating and Assignment

#### 3.3.1. Reservoir Creating with Gaussian Criterion

To estimate an accurate frame-wide transformation, various reservoirs have been used to save matches from different frames. However, existing reservoirs have some disadvantages. Reference [10] presented a FIFO reservoir to preserve matches from *N* continuous frames. When the movements of targets are faint, the reservoir would be filled with matches that are not typical enough for calculating a homography. Reference [12] proposed a reservoir using a voting scheme based on the RANSAC algorithm [28], but it may save some persistent outliers because the RANSAC algorithm is not stable.

**Figure 2:**
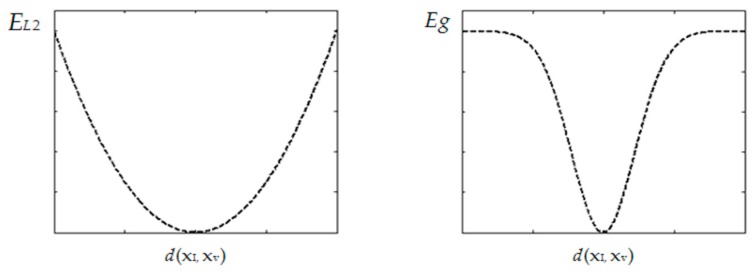
The curves of $L_{2}$ distance (**left**) and Gaussian distance (**right**) for various Euclidean distances $d\left(x_{i},x_{v}\right)$.

Therefore, we propose a novel reservoir for better correspondence preservation. In which, we first calculate the Gaussian distance of each match with the following expression:

(2) $$E_{k}=\exp\left(-\frac{d^{2}\left(H\left(x_{i}\left(k\right)\right),x_{v}\left(k\right)\right)}{\sigma^{2}}-\lambda \cdot C\left(S_{i}\left(k\right),S_{v}\left(k\right)\right)\right)$$

where $x_{i}\left(k\right)$ and $x_{v}\left(k\right)$ are the infrared and the visible feature points in the match, respectively. $d^{2}\left(H\left(x_{i}\left(k\right)\right),x_{v}\left(k\right)\right)$ is the $L_{2}$ distance of the point pair transformed by the current matrix H, and $C\left(S_{i}\left(k\right),S_{v}\left(k\right)\right)$ represents the descriptor similarity calculated by Equation (1). σ represents a range parameter, and λ is a balanced factor which controls the trade-off between spatial and attribute distances of the match. With the Gaussian criterion, we can together consider the matching metric and the homography adaptability of the match. When the reservoir is filled, the K-means algorithm [29] is used to divide all matches into two groups (inliers and outliers) according to their Gaussian distances. For a new match, we will randomly pick one of the outliers and replace it. To show the robustness of Gaussian distance, Figure 2 shows the curves of $L_{2}$ distance ($E_{L2}=d^{2}\left(x_{i},x_{v}\right)$) and Gaussian distance ($E_{g}=1-\exp\left(-d^{2}\left(x_{i},x_{v}\right)∕\sigma^{2}\right)$). We see that using the Gaussian distance makes inlier and outlier division become more easy.

#### 3.3.2. Reservoir Assignment Using Multi-Target Tracking

**Figure 3:**
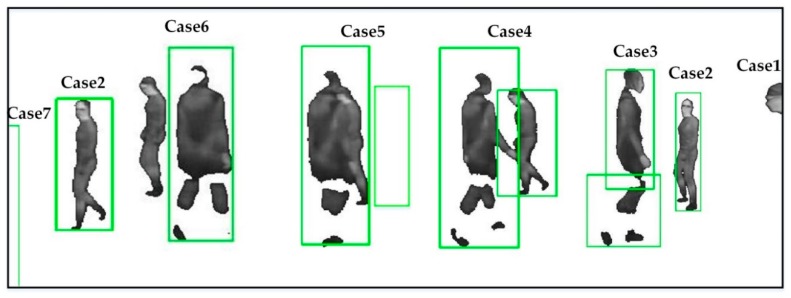
Representative cases (Case: 1–7) of tracking results for the targets with different moving states. The green bounding box shows the output of a tracker. There are seven kinds of moving state, as shown in Case (1–7): entering (Case 1), tracking normally (Case 2), fragmentation (Case 3), occlusion (Case 4), invisible (Case 5), lost tracking (Case 6) and leaving (Case 7).

We need to find the target association in consecutive frames to allot a reservoir for each target. Therefore, a multi-target tracking method [13] is adopted, in which KCF trackers and foregrounds help each other to take tracking decision. The foregrounds are used to get the sizes of moving targets in combination with the outputs of KCF trackers, while KCF trackers are applied to find the association and handle some special cases, such as occlusions. By obtaining the output $RF^{t}$ of each tracker $CF^{t-1}$, the method identifies the moving state of each foreground blob $FOR^{t}$ such as entering, leaving, occlusion and so on. In our work, since infrared targets are more salient, and infrared scenes are not easily influenced by shadow and light changing, we track targets in infrared videos. Some tracking results of the targets with different states are given in Figure 3.

During reservoir assignment, a global reservoir is first created to save matches from all targets. Then, we assign a reservoir to each tracker of the current frame based on the tracking results, which reserves the matches from the corresponding target. This is discussed as follows:

- If only one tracker $CF_{j}^{t-1}$ is associated with a foreground $FOR_{j}^{t}$, it means the target is being tracked normally (Case 2 in Figure 3). At this time, the reservoir of $CF_{j}^{t-1}$ is directly assigned to the tracker $CF_{j}^{t}$, and the matches from the foreground $FOR_{j}^{t}$ are saved in the reservoir.
- More than one tracker $CF_{j}^{t-1}$ may be associated with a foreground $FOR_{j}^{t}$, which could be caused by an occlusion (Case 4) or fragmentation (Case 3). We differentiate the two cases according to the way presented in [13]: if the area of the blob is smaller than the sum of the two trackers in some consecutive frames, it is very likely caused by fragmentation. Otherwise, we assume that two targets are under occlusion. If caused by an occlusion, there are multiple trackers $CF_{j}^{t}$ for the foreground. The reservoir of each $CF_{j}^{t-1}$ is assigned to the corresponding tracker $CF_{j}^{t}$. Each match from the foreground is saved in the reservoir with the tracker closest to the match. If caused by fragmentation, we combine these trackers $CF_{j}^{t-1}$ to produce a new tracker $CF_{j}^{t}$ for the foreground, and merge their reservoirs to bring in a new reservoir for the current tracker. The matches from the foreground are reserved in the reservoir.
- If no tracker $CF_{j}^{t-1}$ is associated with a foreground $FOR_{j}^{t}$, it means a new target is entering the scene (Case 1) or tracking is lost (Case 6). At this moment, a new tracker $CF_{j}^{t}$ is built for the foreground, and a new reservoir is created to keep the point pairs in the foreground. Since the matches in the reservoir may be insufficient to estimate a reliable homography, we introduce part of matches from the global buffer to the reservoir. Next, if caused by tracking failure, the lost reservoir would be merged into the new reservoir, when the lost tracker $CF_{j}^{t-1}$ is recalled.
- If a tracker $CF_{j}^{t-1}$ is not associated with any foreground $FOR^{t}$, it means a target is invisible (Case 5) or leaving (Case 7). In this case, the tracker $CF_{j}^{t-1}$ and its reservoir are saved in some consecutive frames. As presented in [13], if the tracker is associated with a foreground again in these frames, the lost tracker and the reservoir are recalled. Otherwise, the tracker and the reservoir are both removed.

### 3.4. Homography Estimation

To align multiple targets accurately, we estimate a homography for each target. For a foreground blob, one or more matrix is required, whose quantity is the same as the number of reservoirs corresponding to the foreground. Since the matrices from a single frame may be noisy, frame-wide transformations are adopted. Now, we also need a global matrix to ensure registration precision. The current global matrix $Hg_{cur}$ is first computed with all matches in the global reservoir using the RANSAC algorithm [28]. Then, we calculate the overlap error of a transformation using:

(3) $$E\left(M\right)=1-\frac{\Gamma \left(S_{i},M\right)\cap^{\text{​}}S_{v}}{\Gamma \left(S_{i},M\right)\cup^{\text{​}}S_{v}}$$

where M is a transformation matrix, $\Gamma \left(S_{i},M\right)$ is the transformed infrared foreground image, and $S_{v}$ represents the original visible foreground image. After getting the overlap errors $E_{t-1}$ (for the reference global frame-wide $Mg_{t-1}$) and $E_{cur}$ (for the current homography $Hg_{cur}$), we update the global frame-wide matrix with:

(4) $$Mg_{t}=\{\begin{array}{cc} \left(1-\beta \right)*Mg_{t-1}+\beta *Hg_{cur} & if\;E_{cur}<E_{t-1}\\ Mg_{t-1} & otherwise \end{array}$$

where β is an adaptation factor. We used a fixed value in our experiment. After that, we can estimate a global frame-wide homography for the whole scene.

For an infrared foreground blob $B_{i}^{IR}$, we have to confirm its corresponding visible foreground before aligning it. Under this condition, if there is a matched visible foreground during the foreground matching (Section 3.2), the visible foreground is viewed as the correspondence $B_{i}^{vis}$. Otherwise, we transform the infrared foreground with the global matrix $Mg_{t}$. The visible foreground that has the lowest overlap error with the transformed foreground is chosen as the correspondence $B_{i}^{vis}$. After that, we start to estimate the homography of this foreground pair.

Algorithm 1 describes our strategy in detail. If there is only one reservoir for the infrared foreground $B^{IR}$, we first estimate a current homography $Hq^{cur}$ with all matches in the reservoir $R^{cur}$ using the RANSAC algorithm [28], and get its overlap error $E^{cur}$ (Equation (3)). We then discuss the moving state. If tracking normally, the previous frame-wide matrix $Mq^{t-1}$ is viewed as the reference frame-wide $Mq_{ref}^{t-1}$; if fragmentation, there are multiple previous matrices. We consider their average as the reference frame-wide $Mq_{ref}^{t-1}$; if entering or lost tracking, there is no frame-wide homography in the previous matrix group $\left\{Mq^{t-1}\right\}$. In this case, the global matrix $Mg_{t}$ is selected as the reference frame-wide $Mq_{ref}^{t-1}$. Next, we determine the overlap error $E_{ref}^{t-1}$ of $Mq_{ref}^{t-1}$ (Equation (3)). According to $Hq^{cur}$, $E^{cur}$, $Mq_{ref}^{t-1}$ and $E_{ref}^{t-1}$, we last estimate the current frame-wide homography $Mq^{t}$ using Equation (4).

If there is more than one reservoir for the infrared foreground, it means some targets are occluded. At this time, we divide the foreground $B^{IR}$ into M (the number of reservoirs) targets $B_{1}^{IR},B_{2}^{IR},\ldots ,B_{M}^{IR}$. Each target includes the foreground pixels in its tracker, and the pixels that are not in any trackers but are closest to the tracker. For each target $B_{j}^{IR}$, we first compute the current matrix $Hq_{j}^{cur}$ and the current overlap error $E_{j}^{cur}$. Then, we view the corresponding previous frame-wide $Mq_{j}^{t-1}$ as $Mq_{ref}^{t-1}$, and confirm its overlap error $E_{ref}^{t-1}$. According to $Hq_{j}^{cur}$, $E_{j}^{cur}$, $Mq_{ref}^{t-1}$, and $E_{ref}^{t-1}$, we estimate the current frame-wide homography $Mq_{j}^{t}$ for the target (Equation (4)). With the homography estimate algorithm, we can calculate an accurate frame-wide transformation for each target.
**Algorithm 1 Estimating Homography for a Foreground in Current Frame****Input:** Infrared foreground: $B^{IR}$. visible foreground: $B^{VIS}$. Moving state: $F^{IR}$.Reservoir group: $\left\{R^{cur}\right\}$. Previous homography group: $\left\{Mq^{t-1}\right\}$. Global matrix: $Mg_{t}$**Output:** current frame-wide homography group: $\left\{Mq^{t}\right\}$**Proceduce** Homography estimation**  If** $\mathrm{size}\;\left(\left\{R^{cur}\right\}\right)=1$      $R^{cur}\to Hq^{cur}$, $E^{cur}=1-\Gamma \left(B^{IR},Hq^{cur}\right)\cap^{\text{​}}B^{VIS}∕\Gamma \left(B^{IR},Hq^{cur}\right)\cup^{\text{​}}B^{VIS}$      **If**
$F^{IR}=\mathrm{tracking}\;\mathrm{normally}$
$Mq_{ref}^{t-1}=Mq^{t-1}$
**end if;**      **If**
$F^{IR}=\mathrm{fragmentation}$
$Mq_{ref}^{t-1}=\overset{N}{\underset{i=1}{\sum }}Mq_{i}^{t-1}∕N$
**end if**;      **If**
$F^{IR}=\mathrm{entering}\;\mathrm{or}\;\mathrm{lost}\;\mathrm{tracking}$
$Mq_{ref}^{t-1}=Mg_{t}$
**end if;**      $E_{ref}^{t-1}=1-\Gamma \left(B^{IR},Mq_{ref}^{t-1}\right)\cap^{\text{​}}B^{VIS}∕\Gamma \left(B^{IR},Mq_{ref}^{t-1}\right)\cup^{\text{​}}B^{VIS}$      $Hq^{cur},\;E^{cur},\;Mq_{ref}^{t-1},\;E_{ref}^{t-1}\to Mq^{t}$**  Else**      $B^{IR}\to B_{1}^{IR},B_{2}^{IR},\ldots ,B_{M}^{IR}$      **For**
$R_{j}^{cur}\in R_{1}^{cur},R_{2}^{cur},\ldots ,R_{M}^{cur}$
**do**      $R_{j}^{cur}\to Hq_{j}^{cur}$, $E_{j}^{cur}=1-\Gamma \left(B_{j}^{IR},Hq_{j}^{cur}\right)\cap^{\text{​}}B^{VIS}∕\Gamma \left(B_{j}^{IR},Hq_{j}^{cur}\right)\cup^{\text{​}}B^{VIS}$      $Mq_{ref}^{t-1}=Mq_{j}^{t-1}$, $E_{ref}^{t-1}=1-\Gamma \left(B_{j}^{IR},Mq_{ref}^{t-1}\right)\cap^{\text{​}}B^{VIS}∕\Gamma \left(B_{j}^{IR},Mq_{ref}^{t-1}\right)\cup^{\text{​}}B^{VIS}$      $Hq_{j}^{cur}\;,E_{j}^{cur},\;Mq_{ref}^{t-1},\;E_{ref}^{t-1}\to Mq_{j}^{t}$      **End for****  End if****End proceduce**

## 4. Experiments and Results

### 4.1. Experiments

#### 4.1.1. Datasets

To manifest the advantage and generalization of the propose framework, experiments were performed on both near-planar and non-planar scenes, although the framework is presented for non-planar video scene registration. For near-planar scenes, we employed the public LITIV dataset provided by Torabi [22], which includes nine video pairs of 240 × 320 resolution. In these videos, all targets are on near-planar scenes since they were viewed from afar. Furthermore, ground-truth matrices are provided, which were used to produce the results of manual registration. For non-planar scenes, there are few public datasets with videos which contain multiple targets on different depths for IR-visible registration. The OTCBVS dataset provided by Bilodeau [8] is the only one we found. Therefore, four raw video pairs of 360 × 480 resolution in the dataset were used in our experiment. Since the ground-truth is not provided by the dataset, we created our own ground-truth transformations by manually selecting some identifiable points in the infrared and visible images.

#### 4.1.2. Approach Comparison

In the proposed method, different matrices are computed to align multiple targets. Therefore, we first compared our method with a state-of-the-art global registration method [12]. In which, all targets are analyzed together, and a global frame-wide homography is estimated for the whole scene. Detailed, the method directly matched contour points, and saved the feature pairs from each target in a reservoir based on a voting scheme. Next, we also compared the proposed framework with manual ground-truth to show the superiority of our framework. It must be declared that the ground-truth represents the manual registration matrix rather than the reference image. The error of it is not necessarily equal to 0.

In Section 3.4, the global transformation matrix has been estimated to ensure registration accuracy. To validate the robustness of the proposed feature matching strategy and the reservoir, we introduce the proposed global matrix as a comparison in our experiments. Since a global homography is difficult to realize for non-planar registration, the results on OBCTVS dataset cannot directly reflect the robustness of the matching strategy and reservoir. Therefore, the method is applied only on the near-planar database. For fairness, all methods are tested on the same foreground images, and the same error function is adopted to evaluate these methods. Besides, the parameters used by every method were identical in both methods.

#### 4.1.3. Evaluation Metric

Most previous methods [10,12,22] created a pair of binary polygons for each sequence pair by manually selecting some matched points. They used the overlap error between the transformed polygons as evaluation metric. However, there are certain limitations for the metric. First, it only can assess the registration quality for perfectly planar scene. For near-planar or non-planar scene, the metric is useful only when all targets lie on the same depth with the polygons. Otherwise, the polygon overlap error is not competent to measure registration accuracy. Besides, these polygons were built for global registration methods, which calculate only one registration matrix for a frame pair. It could not be adopted in the proposed framework, because more than one matrix may be got simultaneously in our method.

Under the circumstances, we considered the overlap error between the transformed infrared and the corresponding visible foreground images as registration error function, which is defined as:

(5) $$E_{C}\left(M_{1,2,\ldots ,N}\right)=1-\left(\cup_{k=1}^{N}\Gamma \left(B_{k}^{IR},M_{k}\right)\cap^{\text{​}}S_{v}∕\cup_{k=1}^{N}\Gamma \left(B_{k}^{IR},M_{k}\right)\cup^{\text{​}}S_{v}\right)$$

where $M_{k}$ is the transformation matrix of the *k*th infrared target $B_{k}^{IR}$, and $\cup_{k=1}^{N}\Gamma \left(B_{k}^{IR},M_{k}\right)$ is the union set of all transformed infrared targets. $S_{v}$ represents the raw visible foreground image. The error function can measure the alignment of each target for all methods. To be clear, for a frame pair, if there is no target in infrared or visible image, the overlap error of this pair is not considered.

#### 4.1.4. Parameter Settings

There are mainly five parameters in our method: $D_{c}$, $T_{a}$, $\sigma^{2}$, λ and β. Parameters $D_{c}$ and $T_{a}$ control the merging of detected foreground blobs. Only when the nearest distance between two foregrounds is smaller than $D_{c}$ and their area ratio is lower than $T_{a}$, the foreground blobs could be merged. To solve the foreground fragmentation problem but not merge two targets together, the experiments show $D_{c}=50\;\mathrm{pixels}$ and $T_{a}=0.2$ are the best set. Parameter $\sigma^{2}$ is the range factor in calculating the Gaussian distance of each match. Typically, we set $\sigma^{2}=100$. Parameter λ presents the trade-off between the spatial and attribute information of a correspondence. Since we prefer to consider the adaptability of the point pair to the transformation matrix, the parameter λ is set to 0.5. Parameter β is the weight factor for estimating frame-wide homography. To find a middle ground between updating matrix timely and avoiding the local optimum, we initialize β=0.25.

### 4.2. Results

#### 4.2.1. Results for Near-Planar Scenes

**Figure 4:**
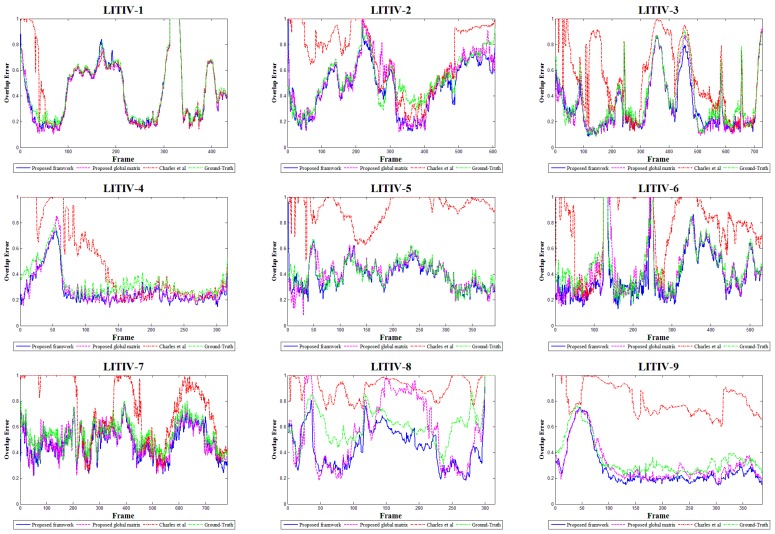
Accuracy comparisons of the ground-truth, the method of [12], the proposed global matrix and our framework using the foreground overlap errors on the LITIV dataset.

Figure 4, Table 1 and Table 2 display the registration errors of all algorithms on near-planar scenes. We find that the proposed global registration matrix usually has lower errors than the registration method presented by Charles [12], although the method also computes a global transformation. This is essentially because our matching strategy and reservoir can provide more accurate correspondences for homography estimation. In the dataset, the sizes of infrared scenes are quite different from those of visible scenes, so the distribution and number of targets may be not consistent between two types of images. The phenomena are very universal throughout each sequence pair. In this case, the proposed feature matching strategy can prevent more mismatches by finding target correspondences, and the reservoir can distinguish inliers and outliers more exactly by using Gaussian criterion.

We also see that the proposed non-planar registration framework succeeds in extracting the proposed global matrix in all video pairs. The situation is caused by the non-planar characteristic of the LITIV dataset. In the dataset, the scenes don’t fully follow the planar assumption, especially for LITIV-8 and LITIV-9. In this case, the framework has a better performance since it can eliminate the influence of varying depths by aligning each target individually.

As shown in these results, our method and the proposed global matrix outperform ground-truth homography in all sequence pairs. This is normal and desirable performance, because there are some disadvantages for the ground-truth. First, the deviation is unavoidable when establishing a ground-truth by manually selecting some point pairs. Second, the ground-truth produces an ideal registration only for a planar scene. However, the moving of the target is not always on one depth plane. Hence, methods that aim to align the current targets have a higher precision. For the proposed method, it is also because the method can implement multi-target registration on non-planar scenes. In addition, we find there are some strong increases in the error curves of these methods, which are caused by the size differences between infrared and visible scenes.

#### 4.2.2. Results for Non-Planar Scenes

**Figure 5:**
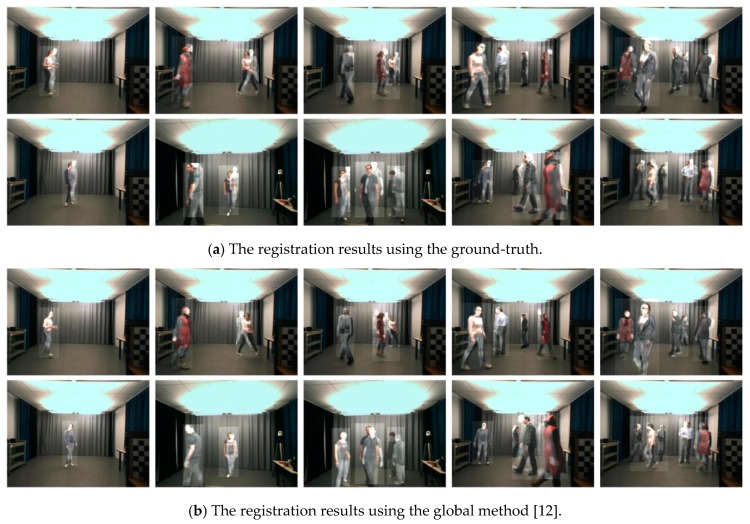

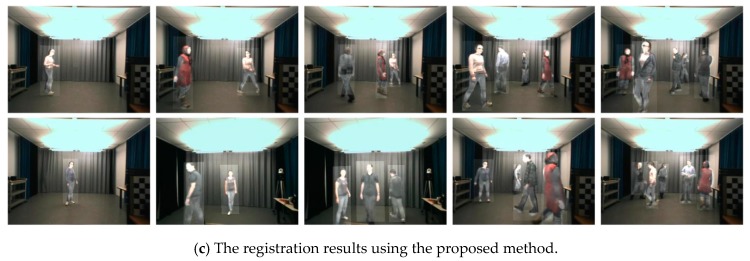
Registration results on some typical frame pairs in the OTCBVS dataset using the manual ground-truth homography, the global method in [12] and the proposed framework, as shown in group (**a**–**c**). Each group contains two sets of images, which is displayed by two rows. Each row contains five images with different numbers of targets.

The registration results on non-planar scenes are shown in Figure 5. The figure contains three groups, which present the results of each method, respectively. There are five images in each row of a group, and the quantities of targets in these images vary from one to five. We superimpose the transformed infrared images on the visible images to display the registration outputs.

We see that the ground-truth cannot align non-planar scenes, even if there is only one target on the scenes. The reason for the situation is that the ground-truth can merely register a depth plane. For any target, if it does not lie on the plane, the ground-truth has no ability to align it. This declares that the global ground-truth matrix is not applicable to non-planar registration. The second group shows the global registration method in [12] can align the scenes which contain one target. This is because the frame-wide homography provided by the method is competent in registering one target, even it moves in three-dimensional space. However, the method fails to complete the registration of multiple targets, since one matrix is not enough to eliminate the influence of depth differences between targets. The last group illustrates that our method always succeeds in aligning all targets on non-planar scenes, regardless of the number of targets. This is no surprise, since our framework provides a frame-wide matrix for each target, and then eliminates the effect of non-planar characteristic. This guarantees that the proposed method could achieve better performance on non-planar multi-target registration than the global method and the ground-truth.

Table 3 and Table 4 give the average and minimum registration errors for all sequences in the non-planar dataset. According to these tables, we observe that our framework significantly reduces the average registration error of each sequence pair (24.14% for Video 1, 35.32% for Video 2, 10.12% for Video 3 and 15.32% for Video 4). Moreover, our method could achieve much lower minimum errors for all studied pairs compared to the method proposed in [12] and the ground-truth. These experimental data reveals that the propose framework is more suitable for non-planar registration than both the automatic and manual global registration methods.

**Figure 6:**
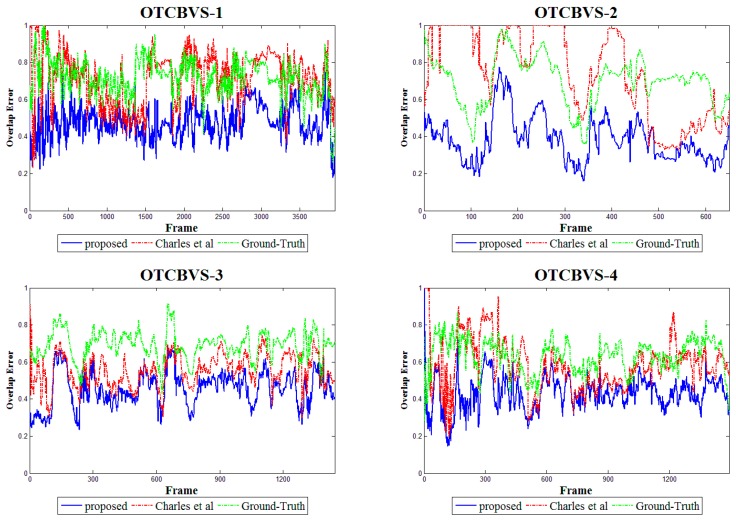
Accuracy comparisons of the ground-truth, the method of [12] and our framework using the foreground overlap errors on the OTCBVS dataset.

In order to reflect the global performance of these methods, we also present error-to-time curves for every video pair, as shown in Figure 6. We see that our method produces the best results, and our error curves are mainly below the curves of two comparison methods. The proposed framework has the ability to achieve a higher precision and stabilize at this level more often than the global registration methods.

**Figure 7:**
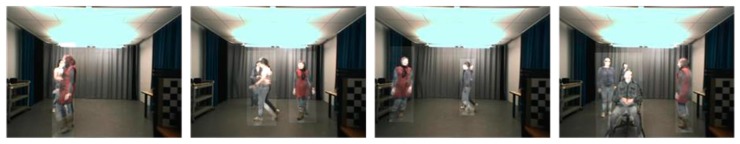
Registration results on some serious occlusion frames in the OTCBVS-1. The results are not accurate since only one homography is computed for multiple targets under the occlusion.

Then, we encounter two questions: why are the registration errors still important? Why are our error curves not smooth? There are two simple answers to these questions. The first one is the occlusion. Our framework is able to process slight partial occlusion. However, when a target is seriously occluded by others, the framework may identify all as one target, and calculate only one homography for these targets. As a result, the registration is not accurate. Serious occlusion is frequently happening throughout each sequence, which leads to the high overlap errors and the fluctuation of registration errors. Registration results on some occlusion frames occurred in OTCBVS-1 are presented in Figure 7.

**Figure 8:**
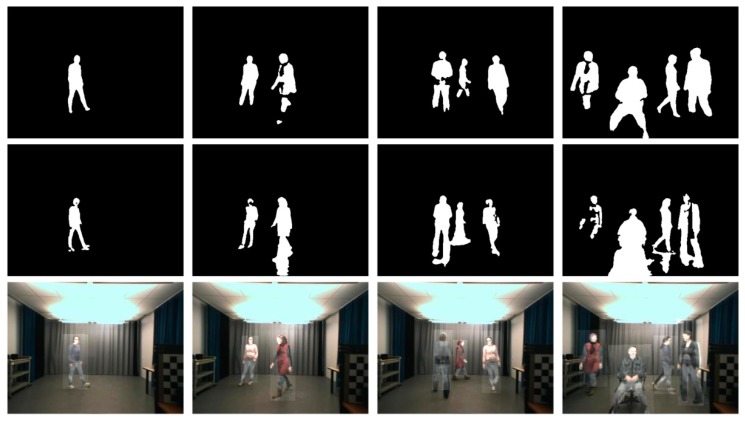
Noisy foreground frame pairs in the OTCBVS dataset and the registration results on these frames. The first row is infrared foregrounds and the second is visible foregrounds. The last row is the registration results on these pairs.

The second answer is the deviation in foreground detection. Actually, foreground detection is imperfect in most infrared and visible images, and foreground fragmentation may occur in some frames. Using the noisy foreground frames, the high overlap errors are obtained in these frames, even if we have estimated an accurate transformation for each target. Therefore, the errors are still important and unstable. Some noisy foreground pairs and the registration results on them are shown in Figure 8. We find that the proposed framework could achieve acceptable registration accuracy in these pairs. This is because the feature matching algorithm and reservoir used by our method ensure that we can get plenty of accurate matches from various frames for registration. This is to say, the proposed method is robust to deviations in foreground extraction.

## 5. Conclusions

In this paper, we have presented a multi-target independent analysis framework to implement non-planar infrared-visible video registration. The method finds foreground correspondences in order to match feature points more robustly and faster. To align multiple targets on different depth planes, we adopted a multi-target tracking method to assign a reservoir for each target. For targets with different moving states, their frame-wide homography is estimated in different ways. Experimental results showed that the proposed framework could precisely register multiple targets on both near-planar and non-planar scenes. It also outperformed a recent state-of-the-art global registration method and the manual ground-truth. Furthermore, the experiments verified that our method is robust to foreground fragmentation.

## Figures and Tables

**Table 1:** Average overlap errors for all sequence pairs of the LITIV dataset (red entries indicate the best results).

Sequence Pair	Ground-Truth	Charles et al.	Proposed Global Matrix	Proposed Framework
LITIV-1	0.4515	0.4850	0.4227	0.4155
LITIV-2	0.5290	0.7196	0.5031	0.4701
LITIV-3	0.3646	0.5545	0.3340	0.3257
LITIV-4	0.3418	0.4838	0.2854	0.2742
LITIV-5	0.4085	0.9130	0.4047	0.3908
LITIV-6	0.4564	0.7852	0.4395	0.3972
LITIV-7	0.5380	0.7524	0.4947	0.4766
LITIV-8	0.6232	0.9118	0.5840	0.4670
LITIV-9	0.3533	0.8091	0.3107	0.2774

**Table 2:** Minimum overlap errors for all sequence pairs of the LITIV dataset (red entries indicate the best results).

Sequence Pair	Ground-Truth	Charles Et. Al.	Proposed Global Matrix	Proposed Framework
LITIV-1	0.1571	0.1308	0.1135	0.1075
LITIV-2	0.1678	0.1971	0.1187	0.1250
LITIV-3	0.0804	0.1093	0.0897	0.0844
LITIV-4	0.1851	0.1746	0.1504	0.1419
LITIV-5	0.1922	0.5075	0.0824	0.1875
LITIV-6	0.1675	0.1923	0.1475	0.1311
LITIV-7	0.3125	0.2343	0.2188	0.2222
LITIV-8	0.3107	0.7281	0.1837	0.1832
LITIV-9	0.2072	0.6036	0.1509	0.1465

**Table 3:** Average overlap errors for all sequence pairs of the OTCBVS dataset (red entries indicate the best results).

Sequence Pair	Ground-Truth	Charles et al.	Proposed
OTCBVS-1	0.7220	0.7176	0.4762
OTCBVS-2	0.6983	0.7423	0.3891
OTCBVS-3	0.6920	0.5537	0.4525
OTCBVS-4	0.6346	0.5839	0.4307

**Table 4:** Minimum overlap errors for all sequence pairs of the OTCBVS dataset (red entries indicate the best results).

Sequence Pair	Ground-Truth	Charles et al.	Proposed
OTCBVS-1	0.2582	0.2300	0.1764
OTCBVS-2	0.3806	0.3263	0.1594
OTCBVS-3	0.4291	0.2981	0.2329
OTCBVS-4	0.2797	0.1978	0.1457
